# Assessment of the *in vitro* metabolic stability of CEP-37440, a selective FAK/ALK inhibitor, in HLMs using fast UPLC–MS/MS method: *in silico* metabolic lability and DEREK alerts screening

**DOI:** 10.3389/fchem.2024.1323738

**Published:** 2024-09-26

**Authors:** Mohamed W. Attwa, Haitham AlRabiah, Ali S. Abdelhameed, Adnan A. Kadi

**Affiliations:** Department of Pharmaceutical Chemistry, College of Pharmacy, King Saud University, Riyadh, Saudi Arabia

**Keywords:** CEP-37440, clearance intrinsic, metabolic stability, DEREK alerts, *in vitro* half-life, P450 metabolic module, LC-MS/MS, StarDrop software package

## Abstract

**Introduction:**

CEP-37440 was synthesized and supplied by the research and development division of Teva Branded Pharmaceutical Products (West Chester, PA, United States). CEP-37440 represents a newly developed compound that exhibits selectivity inhibition of Focal Adhesion Kinase and Anaplastic Lymphoma Kinase FAK/ALK receptors, demonstrating novel characteristics as an orally active inhibitor. The simultaneous inhibition of ALK and FAK can effectively address resistance and enhance the therapeutic efficacy against tumors through a synergistic mechanism.

**Methods:**

The objective of this research was to create an LC-MS/MS method that is precise, efficient, environmentally friendly, and possesses a high level of sensitivity for the quantification of CEP-37440 in human liver microsomes (HLMs). The aforementioned approach was subsequently employed to evaluate the metabolic stability of CEP-37440 in HLMs in an in vitro setting. The validation procedures for the LC-MS/MS analytical method in the HLMs were performed following the bio-analytical method validation guidelines set out by the US-FDA. The AGREE program was utilized to assess the ecological impacts of the current LC-MS/MS methodology.

**Results and Discussion:**

The calibration curve linearity was seen in the range of 1–3000 ng/mL. The inter-day accuracy (% RE) exhibited a range of −2.33% to 3.22%, whilst the intra-day accuracy demonstrated a range of −4.33% to 1.39%. The inter-day precision (% RSD) exhibited a range of 0.38% to 3.60%, whilst the intra-day precision demonstrated a range of 0.16% to 6.28%. The determination of the in vitro half-life (t_1/2_) and moderate intrinsic clearance (C_lint_) of CEP-37440 yielded values of 23.24 min and 34.74 mL/min/kg, respectively. The current manuscript is considered the first analytical study for CEP-37440 quantification with the application to metabolic stability assessment. These results suggest that CEP-37440 can be categorized as a pharmaceutical agent with a moderate extraction ratio. Consequently, it is postulated that the administration of CEP-37440 to patients may not lead to the accrual of dosages within the human organs. According to *in silico* P450 metabolic and DEREK software, minor structural alterations to the ethanolamine moiety or substitution of the group in drug design have the potential to enhance the metabolic stability and safety profile of novel derivatives in comparison to CEP-37440.

## Graphical Abstract

**Figure:**
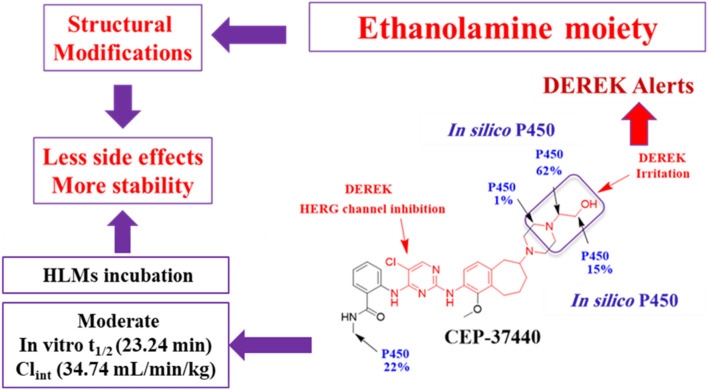

## 1 Introduction

Focal adhesion kinase (FAK), alternatively referred to as PTK2, is a tyrosine kinase enzyme that governs the signaling pathways of integrin and growth factors. Its role encompasses the regulation of cancer cell proliferation, migration, and survival. The expression of FAK is increased in numerous invasive and metastatic malignancies. The FAK protein plays an important role in the progression of tumors and the regulation of multiple cellular processes such as adhesion, motility, invasion, metastasis, survival, angiogenesis, and apoptosis (Golubovskaya, 2014). These functions are closely associated with the transmission of signals through integrins (Schaller et al., 1992). The FAK protein has shown great potential as a viable target for the therapy of cancer. Numerous small molecule FAK inhibitors have been discovered and demonstrated to effectively reduce tumor growth and metastasis in both clinical and preclinical investigations. The overexpression of FAK has been seen in multiple tumor forms, such as breast cancer, prostate cancer, ovarian cancer, lung cancer, and neck cancer. Additionally, FAK has been found to be correlated with a negative prognosis (Béraud et al., 2015). Pharmacologically inhibiting FAK resulted in a decrease in VCAM-1 expression *in vivo*, namely, in tissues, and led to a disruption of metastasis in a mouse model of melanoma (Jeong et al., 2019). In recent years, targeting FAK has emerged as a promising strategy in the field of tumor treatment (Kühn and Armstrong, 2015).

The receptor tyrosine kinase known as anaplastic lymphoma kinase (ALK) is involved in crucial processes such as embryonic and neurodevelopment, as well as tumor formation in humans. Consequently, it has emerged as a significant therapeutic target for the treatment of lung cancer. In recent times, a multitude of preclinical and clinical investigations have uncovered novel functions of ALK in the context of innate immunity immunological evasion, T-cell immunity, and humoral immunity (Kargbo, 2020). The involvement of FAK1 and ALK in the inflammatory activation of endothelial cells produced by Lipopolysaccharide has been documented (Dayang et al., 2021). Furthermore, the identification of FAK as a crucial mediator of the immune response in specific types of cancer has presented compelling evidence supporting the potential benefits of co-administering T-cell immune checkpoint antibodies alongside small-molecule FAK inhibitors (Serrels et al., 2015). Hence, the concurrent inhibition of ALK and FAK can effectively counteract resistance mechanisms and enhance the therapeutic efficacy against tumors through a synergistic effect.

**FIGURE 1:**
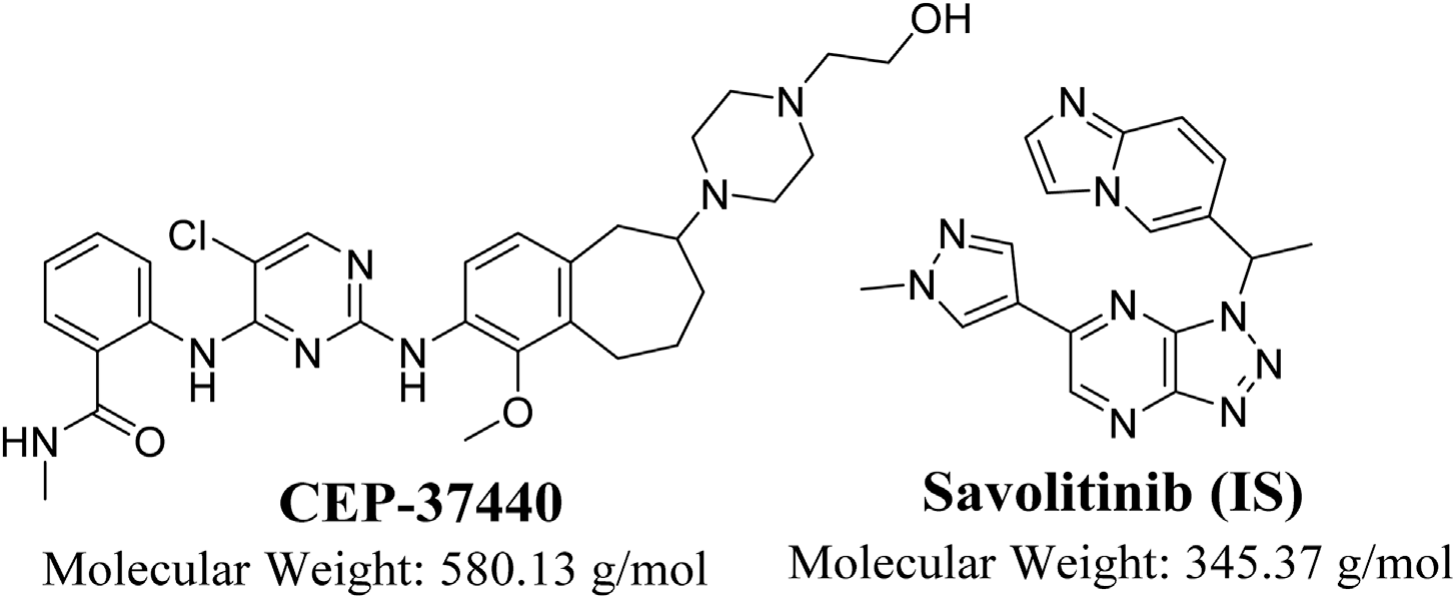
Chemical structure of CEP-37440 (InChIKey: BCSHRERPHLTPEE-NRFANRHFSA-N) and savolitinib (Internal standard; InChIKey: XYDNMOZJKOGZLS-NSHDSACASA-N).

The compound CEP-37440 (Figure 1) was prepared by Teva Branded Pharmaceutical Products R&D, located in West Chester, PA, United States. CEP-37440 is an innovative, discerning, orally bioavailable inhibitor that selectively targets FAK/ALK receptors (Ott et al., 2016). The compound CEP-37440 has the ability to cross the blood-brain barrier and effectively inhibit the formation of brain metastases. The compound CEP-37440 has demonstrated encouraging outcomes in pre-clinical investigations (Ott et al., 2016). The completion of a phase I clinical trial for CEP-37440 occurred in 2015. The major aim of this experiment was to ascertain the safety, maximum tolerated dose (MTD), and tolerability of oral CEP-37440 when delivered on a daily basis to patients diagnosed with metastatic or advanced solid tumors (Sullivan and Planchard, 2016).

The metabolic stability of a pharmacological molecule pertains to its susceptibility to various metabolic processes and is measured by its *in vitro* half-life (t_1/2_) and intrinsic clearance (Cl_int_). The term “half-life” pertains to the temporal interval required for the biotransformation of 50% of the initial pharmacologically active compound. The concept of “intrinsic clearance,” commonly represented as Cl_int_, pertains to the hepatic ability to metabolize a pharmaceutical substance that is circulating in the blood. In contemporary society, there has been an increasing emphasis on the discipline of green analytical chemistry (GAC). This field aims to address the presence of harmful chemicals, decrease energy usage, and minimize the production of waste by employing various analytical methods (Duan et al., 2020; Pena-Pereira et al., 2020). Various measurement approaches have been employed to assess the ecological sustainability of diverse analytical findings, with the objective of achieving these purposes. The study incorporates several methodologies, namely, the Red-Green-Blue (RGB), Analytical Greenness Metric Approach (AGREE), Analytical Eco-Scale (AES), National Green Analytical Procedures Index (GAPI), and Environmental Methods Index (NEMI) (Pena-Pereira et al., 2020). The methodologies AES, NEMI, GAPI, and RGB were dependent on particular values of GAC, as evidenced. The “AGREE” methodology was employed to measure the level of environmental sustainability by evaluating twelve Greenness Assessment Criteria (GAC) criteria and assigning numerical scores to each of them.

Upon completing an extensive review of the available academic literature, it has been determined that there is a notable absence of published research specifically focused on the measurement of CEP-37440 in human liver microsomes (HLMs) with regards to assessing its metabolic stability. Therefore, it is imperative to develop a methodology employing liquid chromatography-tandem mass spectrometry (LC-MS/MS) that is characterized by its expeditiousness and exceptional sensitivity. The utilization of this methodology is crucial for the accurate determination of CEP-37440 in various matrices. The aim of this study was to develop a fast LC-MS/MS method capable of detecting low quantities (as low as 1 ng/mL) of CEP-37440 in a matrix of HLMs. The method was designed to be highly sensitive and specific, while also providing a quick analysis time of less than 2 min. The intended use of this method was to assess the metabolic stability of CEP-37440 in HLMs. Additionally, the results were validated utilizing *in vitro* investigations, namely, metabolic incubations, and *in silico* analysis employing StarDrop software (version 6.6) developed by Optibrium Ltd. (Cambridge, MA, United States).

The evaluation of *in silico* metabolic stability was done using the StarDrop software package (version 6.6), with a specific focus on the *in silico* P450 program. Metabolic lability refers to the quantification of the efficiency of the product production phase within the catalytic cycle of CYP3A4. The presence of metabolic lability can be determined by assessing the composite site lability (CSL) value. A lower CSL indicates a higher probability of enhanced stability. The aforementioned value was employed as initial evidence to support the significance of conducting practical procedures, such as analytical method development and *in vitro* incubation with HLMs, in order to optimize resource allocation and time management (Tyzack and Kirchmair, 2019). The present investigation utilized the LC-MS/MS approach to assess the *in vitro* t_1/2_ and Cl_int_ of CEP-37440 (Marothu Vamsi et al., 2021). Subsequently, the aforementioned factors were employed to compute the *in vivo* metabolism rate utilizing three distinct models, including the parallel tube model, dispersion model, and venous equilibrium model (Houston, 1994; Obach et al., 1997). The estimation of the *in vitro* t_1/2_ and clearance (Cl_int_) of CEP-37440 was conducted using an *in vitro* methodology known as the well-stirred model (Houston, 1994; Obach et al., 1997). This particular model is commonly employed in investigations related to drug metabolism due to its fundamental flexibility. CEP-37440 exhibited a moderate metabolic rate, leading to a favorable *in vivo* bioavailability and a moderate time of action (Alanazi et al., 2019; Alrabiah et al., 2019; Attwa et al., 2022; Darwish et al., 2023). Derek software was utilized to screen for structural alerts inside CEP-37440 chemical structure to support the proposed theory for *in silico* metabolic stability. The goal of the present research was to provide a fast, eco-friendly, extremely sensitive, and selective LC-MS/MS analytical approach for evaluating the metabolic stability of CEP-37440 in the HLMs matrix. The validity of the findings was established through *in vitro* tests employing HLMs incubations and *in silico* analyses conducted with the aid of StarDrop software.

## 2 Materials and methods

### 2.1 Materials

The solvents used in the present LC-MS/MS technique were of HPLC grade. The reference powders utilized in this investigation, specifically CEP-37440 and savolitinib (IS), were of analytical grade. The target analyte, CEP-37440, with a purity of 99.58%, and the IS, savolitinib (IS), with a purity of 99.9%, were acquired from MedChem, a renowned supplier located in Princeton, NJ, United States. The chemicals utilized in this investigation, specifically human liver microsomes (HLMs), ammonium formate (NH_4_COOH), formic acid (HCOOH), and ACN, were acquired from Sigma-Aldrich, a well-regarded supplier headquartered in St. Louis, MO, United States. The HLMs (20 mg/mL) were stored in a refrigeration unit that was maintained at a temperature of −78°C until they were readied for utilization. HPLC-grade water was prepared using an in-house Milli-Q water purification system produced by Millipore (Billerica, MA, United States).

### 2.2 LC-MS/MS instrumental features

The LC-MS/MS approach utilized an isocratic mobile phase and exhibited a fast duration of 2 min. The incorporation of a flow rate (0.4 mL/min), along with a decreased running time (2 min), exerted a notable influence on augmenting the ecologically conscious attributes of the developed technology. Furthermore, the methodology utilized in this research proved a linear relationship throughout a range that extended from 1 to 3,000 ng/mL. The constructed LC-MS/MS approach was validated by doing an *in silico* evaluation of the metabolic stability of CEP-37440 utilizing the *in silico* P450 program (StarDrop’s software) before proceeding with the *in vitro* HLMs incubation (Tyzack and Kirchmair, 2019). The chromatographic separation of CEP-37440 and IS peaks was accomplished using the Acquity UPLC system, specifically the UPH model with a serial number of H10UPH. The mass measurement of the target analyte peaks (CEP-37440 and IS) extracted from the HLMs matrix was conducted using the Acquity TQD MS instrument, specifically the model code TQD and serial number QBB1203. The LC-MS/MS features were tuned to provide effective chromatographic resolution and optimal detection sensitivity of the CEP-37440 and IS analytical peaks. The adjustments made included changes in the mobile phase composition, pH conditions, and polarity of the stationary phase.

The process of separating CEP-37440 and IS involved the utilization of a ZORBAX RRHD Eclipse Plus C18 column (with dimensions of 95 Å, 2.1 × 100 mm, and 3.5 µm) obtained from Agilent Technologies in Santa Clara, CA, United States. This separation was attained by employing isocratic conditions. Mobile phase A was prepared by combining a solution containing 20% of a 10 mM NH_4_COOH solution with a pH of 6.0, which was adjusted using a small amount of formic acid. The mobile phase B composed of 80% ACN and was delivered at a flow rate of 0.4 mL/min.

The mass spectrometry parameters of the TQD MS instrument were adjusted in order to attain optimal sensitivity for detecting the analytical peaks of CEP-37440 and IS. The ESI source was employed in the positive ion mode to facilitate ionization, as the chemical composition of the targets (CEP-37440 and IS) included nitrogen atoms with basic properties that could readily accept protons and generate positively charged ions. The evaporation of droplets in the ESI source was facilitated through the utilization of nitrogen gas (350°C at 100 L/h), which was produced using a nitrogen generator built by Peak Scientific Company (Scotland, United Kingdom). The LC-MS/MS equipment was controlled using the MassLynx package (Version 4.1, SCN 805) as the control software. The MassLynx software package comprises two notable software components, specifically IntelliStart^®^ and QuanLynx. The results generated were subjected to analysis and manipulation using the QuanLynx software. The MS calibration of CEP-37440 (C_30_H_38_ClN_7_O_3_) and IS (C_17_H_15_N_9_) was performed using IntelliStart^®^ software. RF lens voltage, cone gas flow rate, extractor voltage, and capillary voltage were adjusted at 0.1 (V), 100 L/H, 3.0 (V), and 4 kV, respectively. This was accomplished by directly injecting the working concentrations (10 μg/mL) of CEP-37440 and IS into the running mobile phase through the combined feature of the mass analyser. The utilization of the mass analyzer detecting capability of the Triple Quadrupole Detector (TQD) was employed to estimate the levels of CEP-37440 and IS using the technique of multiple reaction monitoring (MRM). The analyte ions CEP-37440 and IS experienced fragmentation into their fragment ions in the collision cell, which is the second quadrupole of the TQD MS analyzer. This fragmentation procedure was enabled by the use of collision gas, specifically argon gas (99.999% at 0.14 mL/min). The mass transition duration for CEP-37440 and IS (parent-to-fragment ions) was 0.025 s. Table 1 presents the MRM characteristics and mass transition attributes pertaining to CEP-37440 and IS (IS).

**TABLE 1:** MRM mode parameters for the estimation of CEP-37440 and IS (IS).

	Time (min)	Retention time	MRM Mass transitions	
Mass spectra segment	0.0 to 1.15	IS (0.9 min)	First *m/z* transition	346.11 → 318.12 (CE^a^: 10, CV^b^: 30)
			Second *m/z* transition	346.11 → 145.16 (CE: 16, CV: 30)
	1.15 to 2.0	CEP-37440 (1.36 min)	First *m/z* transition	580.3 → 419.14 (CE: 28, CV: 44)
			Second *m/z* transition	580.3 → 353.13 (CE: 46, CV: 44)

^a^ Collision energy (eV).

^b^ Cone voltage (V).

### 2.3 *In silico* study of CEP-37440 metabolic lability

The metabolic stability of CEP-37440 was assessed computationally utilizing Optibrium Ltd.’s P450 software integrated within the StarDrop platform (Shin et al., 2011; Tan and Kirchmair, 2014; Hunt et al., 2018; Attwa et al., 2024c). The aforementioned procedure was conducted before the *in vitro* incubation experiment of CEP-37440 with HLMs. The importance of conducting *in vitro* metabolic incubations was validated by the utilization of the results and data obtained through the StarDrop™ software application. The results were further enhanced by the computation of a composite site lability (CSL), which functioned as a measure of the metabolic stability of CEP-37440 (Abdelhameed et al., 2020). The CSL was utilized as a crucial determinant in the assessment of the metabolic stability of CEP-37440. The aforementioned procedure was conducted before commencing the *in vitro* metabolic incubation, with the goal of assessing the requirement for developing the suggested LC-MS/MS system for evaluating the metabolic stability of CEP-37440 (Alrabiah et al., 2019; Attwa et al., 2023). So as to assess the metabolic stability of CEP-37440 (CSL), the CEP-37440s SMILES (CNC(=O)c1ccccc1Nc2c(cnc(n2)Nc3ccc4c(c3OC)CCC[C@@H](C4)N5CCN(CC5)CCO)Cl) was incorporated into the metabolic program. The collection of metabolic instability data for each atom was undertaken in order to calculate the CSL score, as outlined in Equation 1.

CSL=ktotalktotal+kw (1)

where k_w_ is the water formation rate constant.

The CSL protein is observed in both the metabolic landscape and the P450 column dataset. The efficacy of the product production stage in the metabolic cycle of CYP3A4 is quantified by this metric. Therefore, a lower CSL score is indicative of a higher likelihood of enhanced stability (Attwa et al., 2024a).

### 2.4 *In silico* prediction of the toxicity of CEP-37440 using DEREK software

The DEREK software was utilized to do the evaluation of probable toxicity for CEP-37440. Furthermore, the software was utilized to detect structural alerts linked to CEP-37440, in order to propose structural modifications that could potentially reduce the reported toxicity (Marchant et al., 2008; AlRabiah et al., 2020; Attwa et al., 2024b).

### 2.5 CEP-37440 and IS working dilutions

The compounds CEP-37440 and IS exhibited their highest solubility in DMSO at 10 mM and ≥20.83 mg/mL (60.31 mM), correspondingly. Consequently, the CEP-37440 and IS stock solutions, each having a concentration of 1 mg/mL, were solubilized in DMSO. The working solutions (WKs) of CEP-37440 were created at 1 μg/mL, 10 μg/mL, and 100 μg/mL, whereas the IS solution was prepared at 10 μg/mL. The solutions utilized in this study were generated by diluting the stock solutions of CEP-37440 and IS with the mobile phase. The WKs were employed in the fabrication of a total of seven CEP-37440 calibration standards (CSs) and four quality controls (QCs).

### 2.6 Establishing of CEP-37440 calibration standards

Before performing the validation steps for the present LC-MS/MS methodology, HLMs were rendered inactive through exposure to a 2% concentration of dimethyl sulfoxide (DMSO), an organic solvent. The deactivation procedure was executed over 5 min, maintaining a 50°C. The primary aim of the deactivation phase was to mitigate the potential impact of HLMs on the concentrations of the analytes, specifically CEP-37440 and IS, by the inhibition of their metabolic pathways (Fouin-Fortunet et al., 1986; Busby et al., 1999; Störmer et al., 2000). To assess the metabolic stability of CEP-37440, a specialized matrix was created specifically for HLMs. The experimental protocol involved the dilution of 30 µL of inactivated HLMs to 1 mL with the metabolic buffer that consisted of a solution containing 0.1 M sodium phosphate at a pH of 7.4, supplemented with 1 mM NADPH as the enzyme cofactor, and 3.3 mM MgCl_2_. The objective of this dilution was to repeat the circumstances of the *in vitro* incubation of CEP-37440 and HLMs.

The calibration standards (CSs) for CEP-37440 were prepared by sequentially diluting the WKs (WK2 and WK3) of CEP-37440 using a deactivated HLMs matrix. The technique described above resulted in the production of seven separate concentration standards (CSs) at 1, 15, 50, 300, 500, 1,500, and 3,000 ng/mL, respectively. Furthermore, four QCs were produced at 1 ng/mL (LLOQ), 3 ng/mL (representing the lower QC; LQC), 900 ng/mL (representing the medium QC; MQC), and 2,400 ng/mL (representing the higher QC; HQC). Throughout the duration of this experiment, the concentration of the HLMs matrix was continually upheld at a level beyond 90% to counteract any potential effects arising from matrix dilution that could arise throughout the *in vitro* incubation period. QCs were employed as typical samples with unknown concentrations. The concentrations of the QCs were determined using a regression equation derived from the simultaneous injection of CSs of CEP-37440. A solution of IS WK, with a volume of 100 µL and a concentration of 10,000 ng/mL, was added to 1 mL of both CEP-37440 CSs and QCs as an internal standard (IS).

### 2.7 Extraction of CEP-37440 and IS from the HLMs matrix

The protein precipitation process was successfully employed to remove CEP-37440 and IS from the HLMs matrix. The procedure entailed utilizing ACN, an organic solvent, as both a precipitant for proteins and a quencher for metabolic reactions in the HLMs matrix. As a result, 2 mL of ACN was added to both the CEP-37440 CSs and QCs. The samples were exposed to continuous agitation for 5 min in order to help the extraction of CEP-37440 and IS from the precipitated proteins. Subsequently, a centrifugation process was performed for a period of 12 min utilizing a temperature-controlled centrifuge set at 4°C and rotating at a speed of 14,000 rpm. The objective of the centrifugation stage was to facilitate the separation of proteins and promote clarity of the supernatants. To ensure the suitability and dependability of the samples for utilization in the LC-MS/MS instrument, a filtration protocol was executed on all incubates employing a 0.22 µm syringe filter. The filtered extracts were subsequently placed into specialized vials for loading into the LC-MS/MS system. To ensure the lack of interference from the HLMs matrix ingredients during the elution time of CEP-37440 and IS, the experimental procedure described earlier was employed to generate negative control and positive control samples. The experimental sample identified as the positive control comprised of IS within the HLMs matrix. The process of establishing a calibration curve for CEP-37440 entailed graphing the designated outcomes of CEP-37440 on the horizontal axis, while the vertical axis represented the peak area ratio of CEP-37440 to IS. The evaluation of the linearity range of the CEP-37440 CSs that were created involved the examination of various validation characteristics and the determination of the linear regression equation (y = ax + b; *r* ^2^), as a is slope, b is *y*-intercept, and *r* ^2^ is coefficient of variation, using the LC-MS/MS method.

### 2.8 Validation of the current UPLC-MS/MS method

The validation process of the UPLC-MS/MS technology encompassed the assessment of various critical parameters, including precision, linearity, sensitivity, extraction, accuracy, recovery, stability, specificity, and matrix effect. The validation methodology followed the analytical method validation criteria outlined in the guidelines issued by the FDA (Smith, 2012; Meesters and Voswinkel, 2018).

#### 2.8.1 Specificity

The specificity of the current UPLC-MS/MS approach was assessed by injecting six sets of blank human liver microsomes (HLMs) samples after the extraction operation was performed. The purified extracts were fed into the UPLC-MS/MS apparatus for analysis in order to assess whether any interference was observed with the chromatographic peaks produced by the matrix at the identical retention time as the CEP-37440 or IS peaks. Following that, the acquired data were compared with spiked HLMs matrix samples containing the specific analytes of interest, namely, CEP-37440 and IS. The MRM method was employed to mitigate the carryover effect caused by the targets, CEP-37440 and IS, in the TQD analyzer. The validation of this was attained by analyzing the negative control samples of HLMs, which did not include CEP-37440 and IS. Subsequently, the resulting effects were observed.

#### 2.8.2 Sensitivity and linearity

The evaluation of the linearity and sensitivity of the UPLC-MS/MS technology in question required the creation of 12 calibration sets (7 calibration standards) and were used to quantify CEP-37440 in the HLMs matrix. The assessment was completed over the course of a single day. Following that, the calibration standards were treated as unknowns and then back calculated using the regression equation that was obtained from each calibration curve. The determination of the limit of quantification (LOQ) and limit of detection (LOD) required the computation of the slope of the calibration curve and the standard deviation of the intercept, as stated in Equations 2, 3 correspondingly (González and Alonso, 2020).

LOD=3.3*SD of the interceptSlope (2)

LOQ=10*SD of the interceptSlope (3)

The linearity of the LC-MS/MS approach was evaluated through the determination of the coefficient of variation (*r* ^2^) and the application of the least squares method (y = ax + b).

#### 2.8.3 Accuracy and precision

The evaluation of accuracy and precision in the present LC-MS/MS technique entailed conducting many tests over a series of consecutive days. To assess the precision and accuracy of the CEP-37440 QCs, a series of six sets were analyzed across a 3-day period to evaluate inter-day performance. Furthermore, a total of twelve sets were conducted during a single day in order to assess the accuracy and precision within the same day. The accuracy and precision of the LC-MS/MS approach were assessed by measuring them using the metrics of percentage relative error (% RE) and percentage relative standard deviation (% RSD), respectively. The estimated values were derived using Equations 4, 5, correspondingly.

% Error=average computed conc.—supposed conc. supposed conc. *100 (4)

% RSD=SD Mean (5)

#### 2.8.4 Matrix effect and extraction recovery

The evaluation of the influence of the HLMs matrix on the ionization of CEP-37440 and IS was conducted by segregating the samples into two separate cohorts. One calibration curve was established and linear regression equation was generated to be used in the estimation of CEP-37440 in the HLMs matrix. The HLMs matrix of Group 1 was expanded by including the CEP-37440 LQC at 3 ng/mL and IS at 1,000 ng/mL. On the other hand, group 2 was prepared by using the mobile phase in place of the metabolic HLMs matrix. Equation 6 was utilized to calculate the normalized matrix effect (ME) of the internal standard (IS), whilst Equation 7 was employed to determine the ME for CEP-37440 and IS.

IS normalized ME=ME of CEP−37440 ME of IS IS (6)

ME of CEP−37440 or IS=mean peak area ratio Group 1Group 2×100 (7)

The present study conducted an evaluation of the efficacy of the CEP-37440 extraction method from the HLMs matrix, as well as an investigation into the impact of HLMs on the degree of CEP-37440 ionization. This was accomplished by delivering four QCs samples through injection. The validation of protein precipitation as the chosen extraction methodology for CEP-37440 and IS was verified by analyzing six sets of four QCs in the HLMs (B), and afterwards comparing them with four QCs samples generated in the mobile phase (A). The determination of the extraction efficiencies of CEP-37440 and IS was accomplished by calculating the percentage of B relative to A, multiplied by 100.

#### 2.8.5 Stability

The stability of CEP-37440 in the matrix of HLMs was evaluated by assessing the concentration of CEP-37440 at two distinct quality control levels, namely, the low quality control (LQC) and high quality control (HQC) levels. The purpose of this investigation was to assess the reliability and consistency of the LC-MS/MS approach across different storage conditions. The estimation was performed by conducting five replicate measurements under diverse laboratory settings, encompassing storage for both brief and extended periods, storage in the auto-sampler, and subjecting the samples to three cycles of freezing and thawing. In order to evaluate the freeze-thaw stability, the LQC and HQC samples were subjected to a total of three freeze-thaw cycles. Each iteration of the experiment consisted of submitting the samples to freezing conditions at a temperature of −80°C in a refrigerator, followed by thawing at ambient temperature before proceeding with subsequent procedures. In the context of short-term stability studies, the LQC and HQC samples were subjected to processing and subsequent analysis after being stored at ambient temperature on the laboratory benchtop for a duration of 8 h. To evaluate the enduring stability, the spiked HLMs matrix was subjected to a refrigeration process at −80°C for 3 months before commencing the analysis. The stability of the manufactured samples in the auto-sampler was evaluated by exposing them to a storage duration of 24 h at a temperature of 10°C, and afterwards injecting them into the LC-MS/MS instrument.

### 2.9 *In silico* evaluation of the greenness of the current LC-MS/MS method

The perception of green analytical chemistry (GAC) has gained prominence in current academic discussions as a significant scientific paradigm. The main goal of this initiative is to effectively address or diminish the occurrence of potentially harmful substances, diminish the production of waste, and minimize the utilization of energy across several analytical procedures (Duan et al., 2020; Pena-Pereira et al., 2020). To assess this specific aspect, various metric approaches have been employed to assess the ecological sustainability of different analytical assessments. These approaches include the National Environmental Methods Index (NEMI),Red-Green-Blue (RGB), Analytical Greenness Metric Approach (AGREE), Analytical Eco-Scale (AES), and Green Analytical Procedures Index (GAPI) (Pena-Pereira et al., 2020). Among the above-mentioned approaches, NEMI, AES, GAPI, and RGB rely on a restricted range of GAC values. The approaches utilized the “AGREE” methodology to forecast the level of greenness. This involved assessing the greenness by considering the scores of 12 GAC criteria.

### 2.10 *In vitro* estimation of the metabolic stability of CEP-37440

The estimation of Cl_int_ (clearance) and the *in vitro* half-life (t_1/2_) of CEP-37440 were determined by evaluating the residual ratio of CEP-37440 after undergoing *in vitro* metabolism. This was accomplished by utilizing an active HLMs incubation matrix comprising of NADPH, an enzyme cofactor, and MgCl_2_. The experimental protocol for *in vitro* metabolic incubation consisted of four consecutive steps (Abdelhameed et al., 2019; Kadi et al., 2019). During the preliminary phase of the study, a 1 µL volume of CEP-37440 was exposed to preincubation with a matrix of metabolic HLMs. The method described above was carried out at a temperature of 37°C for a period of 10 min, utilizing a water bath that was under thermostatic control. In the beginning stage, a solution containing a concentration of 1 mM of NADPH was added to each individual sample. Following this, the specimens were placed in a thermostatic shaking water bath, where they were maintained at a consistent temperature of 37°C. In the third phase of the experiment, a 100 µL volume of IS solution (1,000 ng/mL) was delivered before the introduction of ACN, which was used as a quenching solvent. The objective of employing this methodology was to attain a uniform level of the IS and mitigate the potential influence of metabolic responses on the concentration of the IS. During the fourth phase, referred to as termination, 2 mL of ACN was added to all samples at specific time intervals. The intervals that were recorded in this investigation included the following time points: 0, 2.5, 7.5, 15, 20, 30, 40, 50, 60, and 70 min. The purpose of this step was to inhibit the enzymatic pathways and cause the precipitation of any surplus proteins. This step is crucial in the extraction process of CEP-37440 and IS, as described in Section 2.6. In order to evaluate the influence of incubation circumstances or matrix components on the concentration of CEP-37440 during the *in vitro* incubation studies, a negative control incubation was prepared. This control involved incubating CEP-37440 with HLMs without NADPH, following the aforementioned procedure.

The determination of the residual concentration of CEP-37440 was accomplished by employing the regression line equation generated from the concurrent loading of calibration standards containing CEP-37440. The process of constructing the stability curve for CEP-37440 entailed the graphical depiction of specific time points, spanning from 0 to 70 min, on the *x*-axis. A graphical representation was generated to depict the relative proportion of CEP-37440 concentration that persists on the *y*-axis, in relation to the starting concentration at time zero (100%) that represented the first constructed curve. Following this, the time period from 0 min to 40 min of the first constructed curve was selected in order to create the second logarithmic curve that accurately reproduces the underlying patterns. To achieve this objective, a graphical depiction was generated by plotting the natural logarithm of CEP-37440 concentrations (ln) against the time intervals of metabolic incubation, ranging from 0 to 40 min. The rate constant pertaining to the metabolic stability of CEP-37440 was ascertained by evaluating the gradient of the preceding curve. The slope obtained from the data was subsequently utilized to compute the *in vitro* half-life by employing the formula *in vitro* half-life = natural logarithm of 2 divided by the slope (McNaney et al., 2008). The calculation of the CEP-37440 Cl_int_ (mL/min/Kg) was performed by taking into account the liver tissue mass (26 g) per kilogram of body weight and the HLMs (45 mg) per gram of liver tissue, as outlined in Equation 8 (Słoczyńska et al., 2019).

$${\text{Cl}}_{\mathrm{int},}=0693\;\times \;\frac{1}{{\;t_{1/2}\;}_{½}\left(\min.\right)}\;\times \;\frac{\text{mL}\;\text{incubation}}{\text{mg}\;\text{protein}}\;\times \frac{\text{mg}\;\text{microsomal}\;\text{proteins}}{g\;\text{of}\;\text{liver}\;\text{weight}}\times \;\frac{g\;\text{liver}}{\text{Kg}\;b.\;w.}$$ (8)

## 3 Results and discussions

### 3.1 LC–MS/MS methodology development

The research examined various stationary phases, which included both nonpolar and polar properties. The experimental setup involved the utilization of a hydrophilic interaction liquid chromatography (HILIC) DIOL column (2.1 × 100 mm, 1.7 µm), which was purchased from Fortis™ Technologies Ltd. located in Neston, United Kingdom. Additionally, a polar imidazole column measuring 2.1 mm × 50 mm and having a particle size of 1.8 µm was acquired from Sepax technologies based in Newark, DE, United States. However, the polar columns employed in this investigation demonstrated certain limits in their capacity of effectively separate and retain CEP-37440 and IS. Nevertheless, the C18 column, employing a reversed stationary phase, yielded the most favourable outcomes. Despite the utilization of a C8 column in the LC-MS/MS approach to attain chromatographic retention of CEP-37440 and IS, it was seen that the aforementioned analytes showed insufficient separation of base peaks, prolonged elution times, and peak tailing. The analysis of CEP-37440 revealed the presence of peak tailing and extended duration of analysis when studying a solution containing 10 mM NH_4_COOH with a pH greater than 6.0. Furthermore, the utilization of a pH value below 6.0, specifically at 3.2, employing a solution composing of 0.1% formic acid in water, led to overlapping of analytical peaks and poor resolution. Once the concentration of ACN exceeded 80%, it became impossible to differentiate among the analytical peaks of CEP-37440 and IS. On the other hand, a reduction in the ratio led to longer elution times.

**FIGURE 2:**
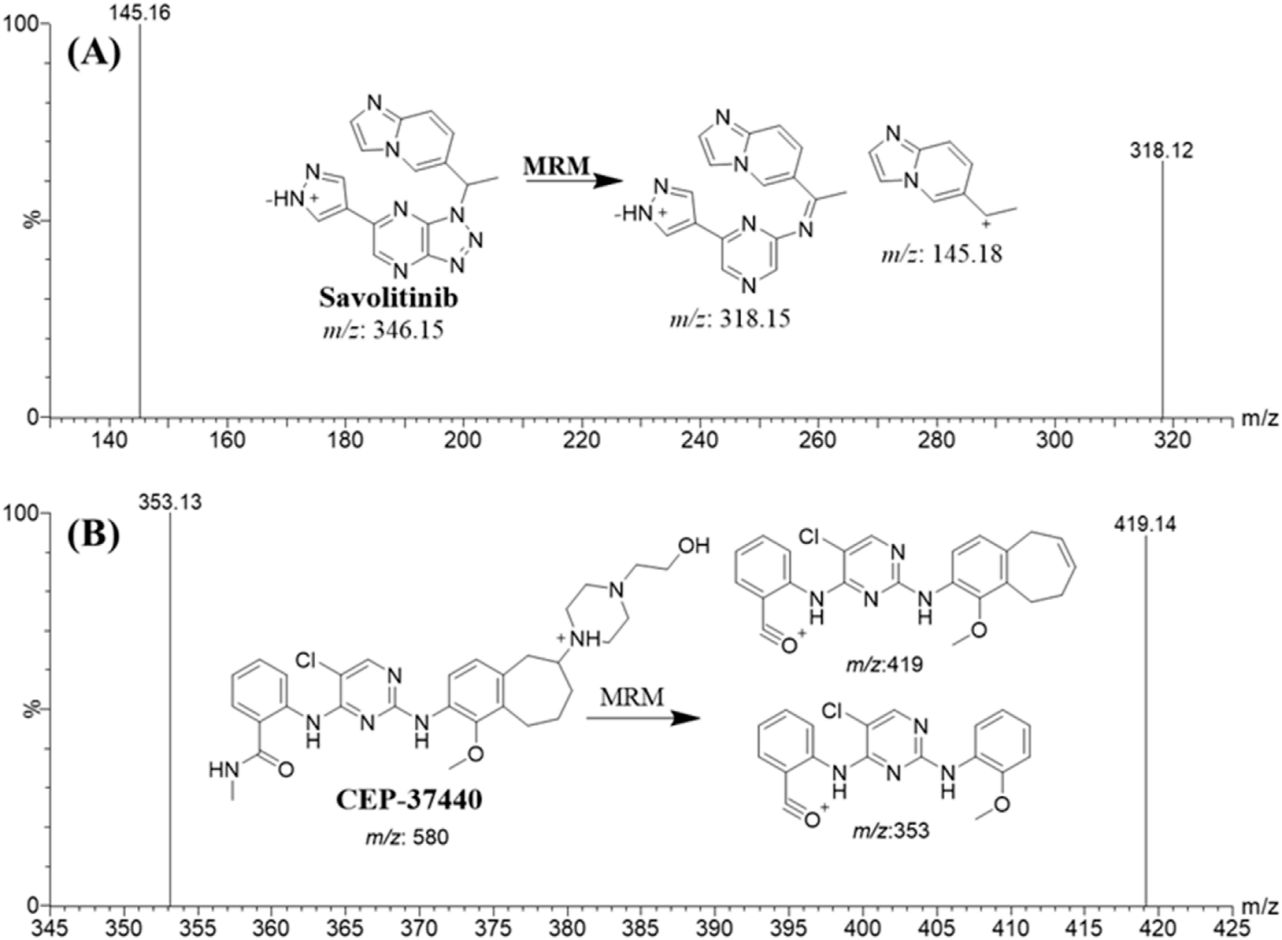
MRM spectrum of IS [M + H]^+^ **(A)** and CEP-37440 [M + H]^+^ **(B)**. The predicted fragmentation behaviours are explained.

The column used in this study was the ZORBAX RRHD Eclipse Plus C18 column that exhibited favourable outcomes in terms of retention time and chromatographic peak shape. The separation of CEP-37440 and IS was achieved in the LC-MS/MS technique by the utilization of a chromatographic apparatus employing an isocratic mobile phase. The separation was conducted at a flow rate of 0.4 mL/min for a duration of 2 min. The calibration curve for CEP-37440 displayed linearity in the concentration range of 1–3,000 ng/mL. With the aim of enhancing the sensitivity of the LC-MS/MS method, the utilization of the MRM detection mode was implemented for the target of detecting and quantifying CEP-37440 and IS. The purpose of this action was to improve the sensitivity and selectivity of the LC-MS/MS methodology that had been previously created. The aim of this method was to minimize any potential interference that was generated from the HLMs matrix components, as illustrated in Figure 2.

**FIGURE 3:**
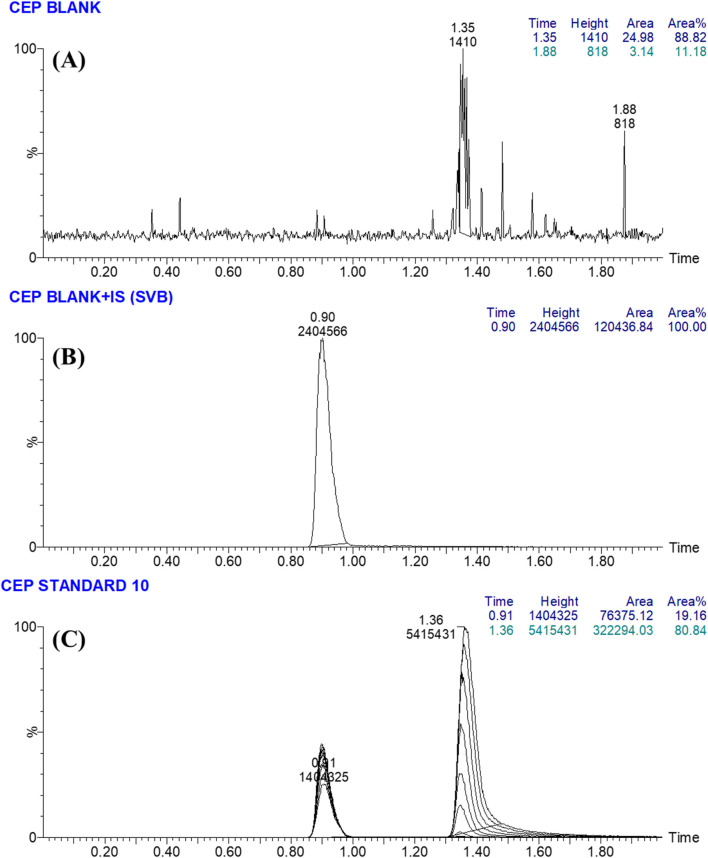
**(A)** The negative control sample consisting of the HLMs matrix did not show any peaks at the same elution times of CEP-37440 and Savolitinib (IS). **(B)** The MRM chromatogram of blank HLMs spiked with IS at 1,000 ng/mL. **(C)** The MRM mass chromatograms of the 3 QCs (3, 900, and 2,400 ng/mL) for CEP-37440 were superimposed, allowing for the identification of the CEP-37440 peaks at 1.36 min. Additionally, the chromatogram displayed a peak corresponding to IS (1,000 ng/mL), appearing at 0.90 min.

The utilization of IS as the internal standard (IS) was implemented in the estimation of CEP-37440, employing the well-developed LC-MS/MS technique for three distinct objectives. It is crucial to emphasize that protein precipitation, as an extraction technique, can be successfully used for both CEP-37440 and IS, resulting in a notable enhancement in productivity. The average yield of CEP-37440 was found to be 99.19% ± 2.65% (Relative Standard Deviation: 2.67), while the yield of IS was seen to be 100.88% ± 2.31% (Relative Standard Deviation: 2.29). Furthermore, it was observed that the eluted peaks corresponding to the IS (0.90 min) and CEP-37440 (1.36 min) analytes were successfully achieved within a time period of less than 2 min. The observed result provides empirical support for the efficacy of the LC-MS/MS technique described in this investigation, since it facilitates rapid separation. This methodology facilitates a substantial reduction in the length of the procedure to a just 2 min, while also utilizing a decreased amount of ACN, so promoting the preservation of the environment. Moreover, it is essential to highlight that the two drugs (CEP-37440 and IS), did not prescribed together to the same patient in a certain medical case. Therefore, the current LC-MS/MS technology can be utilized for pharmacokinetic research and therapeutic drug monitoring (TDM) of CEP-37440. A minimal amount of carry-over was observed in the MRM chromatograms of HLMs controls for CEP-37440, both in the negative (Figure 3A) and positive (Figure 3B) samples. Figure 3C displays the MRM chromatograms of CEP-37440 QSs at concentrations of 3, 900, and 2,400 ng/mL, together with IS at 1,000 ng/mL. The chromatograms have been superimposed in order to facilitate comparison.

### 3.2 Validation features of the LC-MS/MS method

#### 3.2.1 Specificity

The validity of the LC-MS/MS technique was verified by successfully distinguishing the analytical peaks of CEP-37440 and IS, as illustrated in Figure 3. Moreover, it was revealed that there was no detectable interaction among the analytical peaks of targets (CEP-37440 and IS) and the matrix constituents of the HLMs. A minimal level of residual impact from CEP-37440 was observed in the MRM mass chromatograms of the negative control.

#### 3.2.2 Sensitivity and linearity

**FIGURE 4:**
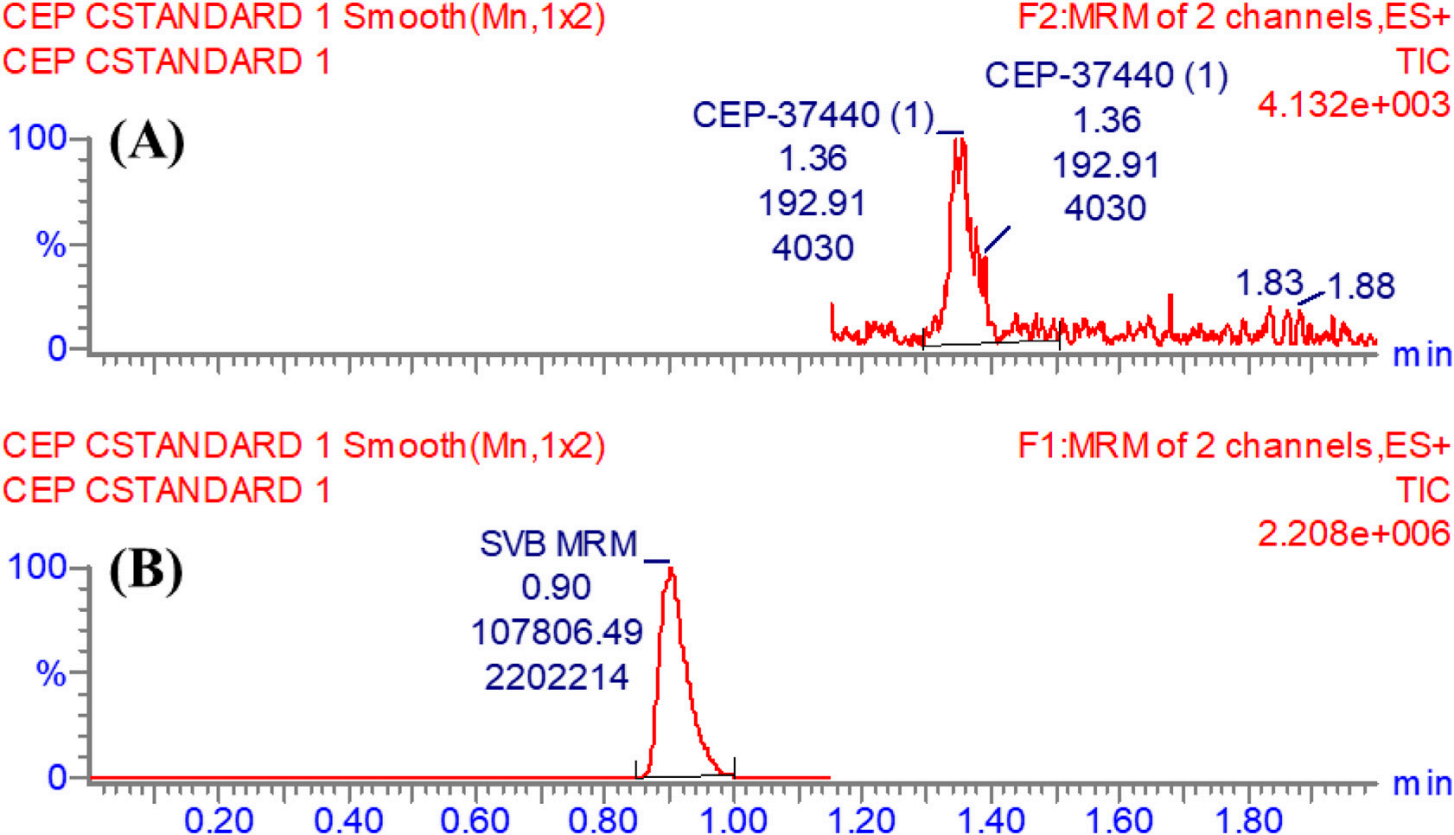
**(A)** The LOQ for CEP-37440 standard (1 ng/mL) demonstrates the sensitivity of the LC-MS/MS technology currently employed. **(B)** IS chromatographic peak at 1,000 ng/mL in HPLC vial while the concentration within the column was measured to be 5 ng/mL.

The linearity range of the LC-MS/MS technique was evaluated by examining a concentration limit of 1–3,000 ng/mL. The successful completion of this work entailed incorporating seven CEP-37440 CSs into the HLMs matrix and subsequently analyzing them as unidentified variables. The linear regression equation derived from the data may be represented as y = 1.4212x – 1.972, with a *r* ^2^ value of 0.9994. The development of the CEP-37440 calibration curve involves the application of mathematical weighting techniques, notably utilizing the reciprocal function (1/x), to account for the substantial variability seen in the CSs. The relative standard deviations (RSDs) of the six replicates (CSs) were found to be less than 3.61%, as indicated in Table 2. The limits of detection (LOD) and limits of quantification (LOQ) were determined to be 0.31 ng/mL and 0.94 ng/mL, respectively, as depicted in Figure 4.

**TABLE 2:** A concise overview of the back-calculation outcomes for six repeats of acalabrutinib (CEP-37440), denoted as CSs.

CEP-37440 (ng/mL)	Mean	SD	RSD (%)	Accuracy (%)	Recovery
1.0	0.96	0.02	2.18	−4.33	95.67
15.0	14.58	0.27	1.84	−2.80	97.20
50.0	51.81	1.03	1.98	3.61	103.61
300.0	292.89	6.48	2.21	−2.37	97.63
500.0	495.33	8.38	1.69	−0.93	99.07
1,500.0	1,503.53	7.68	0.51	0.24	100.24
3,000.0	3,026.59	21.46	0.71	0.89	100.89
% Recovery					99.19 ± 2.65

#### 3.2.3 Accuracy and precision

The assessment of the accuracy and precision of the LC-MS/MS approach was conducted by running 12 sets, each consisting of four QCs samples, in only 1 day’s time. In addition, a total of six sets were carried out, with each set consisting of four samples for quality control. These sets were conducted continuously over a span of three consecutive days. The results obtained were determined to fall in the allowed range as indicated by the standards of validation set out by the Food and Drug Administration (FDA) (González and Alonso, 2020). The LC-MS/MS approach developed in this study exhibited inter-day and intra-day precision and accuracy values within the range of −2.33%–3.22% and −4.33%–6.28%, respectively, as shown in Table 3.

**TABLE 3:** Precision and accuracy of the developed LC-MS/MS approach.

CEP-37440 (ng/mL)	Intra-day				Inter-day			
QCs	1	3	900	2,400	1	3	900	2,400
Mean	0.96	3.02	912.54	2,388.17	0.98	3.10	923.74	2,404.06
SD	0.02	0.19	10.53	3.79	0.04	0.03	11.54	9.14
Precision (%RSD)	2.18	6.28	1.15	0.16	3.60	1.04	1.25	0.38
Accuracy (% RE)	−4.33	0.56	1.39	−0.49	−2.33	3.22	2.64	0.17
Recovery (%)	95.67	100.56	101.39	99.51	97.67	103.22	102.64	100.17

#### 3.2.4 The application of HLMs does not demonstrate any discernible impact on the extraction and recovery of CEP-37440 in the LC-MS/MS methodology MS/MS method

The effectiveness of the chosen protein precipitation extraction technique for the current LC-MS/MS approach was assessed through six replicate measurements, consisting of four QCs samples, within a matrix of HLMs. Subsequently, the obtained data were juxtaposed with QCs samples that had been generated using the mobile phase. The study’s results demonstrated a statistically significant improvement rate for CEP-37440, with an average value of 99.19% ± 2.65% (RSD < 2.67%). Similarly, the extraction recovery rate of IS was found to be noteworthy, exhibiting an average value of 100.88% and a SD of 2.31% (with a RSD below 2.29%). The examination of two sets of injected samples from HLMs matrix yielded findings indicating the absence of any observable impact on the production of ions linked to CEP-37440 or IS. Group set 1 was formed by combining CEP-37440 (LQC, 3 ng/mL) and IS (LQC, 1,000 ng/mL), whereas sample group 2 was created by replacing the HLMs matrix with the mobile phase. The HLMs incorporated the compound CEP-37440 and the substance IS, resulting in a matrix effect (ME) of 100.49% ± 0.94% and 100.23% ± 2.67%, respectively. The researchers calculated the normalized ME of the IS to be 1.0, which is within the permitted range specified by the rules established by the FDA. Based on the results of the experiment, it can be inferred that the HLMs matrix does not have a substantial influence on the ionization of IS or CEP-37440.

#### 3.2.5 CEP-37440 exhibits good stability in the HLMs matrix and stock solution

The evaluation of the stability of CEP-37440 in its initial solution, particularly in DMSO, and in the incubation medium comprising HLMs, demonstrated that the most favorable stability was achieved by storing CEP-37440 in DMSO at −80°C for a duration of 28 days. The obtained analytical findings indicate that the relative standard deviation (RSD%) for each sample of CEP-37440 was found to be less than 3.31% under different storage circumstances, as illustrated in Table 4. No significant decrease in the concentration of CEP-37440 was seen following exposure to several storage circumstances, such as storage in the auto-sampler, short-term storage, experiencing three freeze-thaw cycles, and long-term storage. The results of the experiment revealed that the CEP-37440 molecule had a substantial level of stability.

**TABLE 4:** **T**he stability analysis of CEP-37440.

Stability features	3.0	2,400.0	3.0	2,400.0	3.0	2,400.0	3.0	2,400.0
	Mean		SD		RSD (%)		Accuracy (%)	
Long-Term Stability	2.95	2,394.79	0.03	1.43	0.85	0.06	−1.78	−0.22
Auto-sampler Stability	3.02	2,411.88	0.04	13.39	1.16	0.56	0.56	0.49
Short-Term Stability	3.02	2,394.87	0.10	9.37	3.31	0.39	0.67	−0.21
Freeze–thaw Stability	2.90	2,389.56	0.05	3.29	1.82	0.14	−3.33	−0.44

### 3.3 The assessment of the environmental sustainability of the current LC-MS/MS approach by employing the AGREE program

**FIGURE 5:**
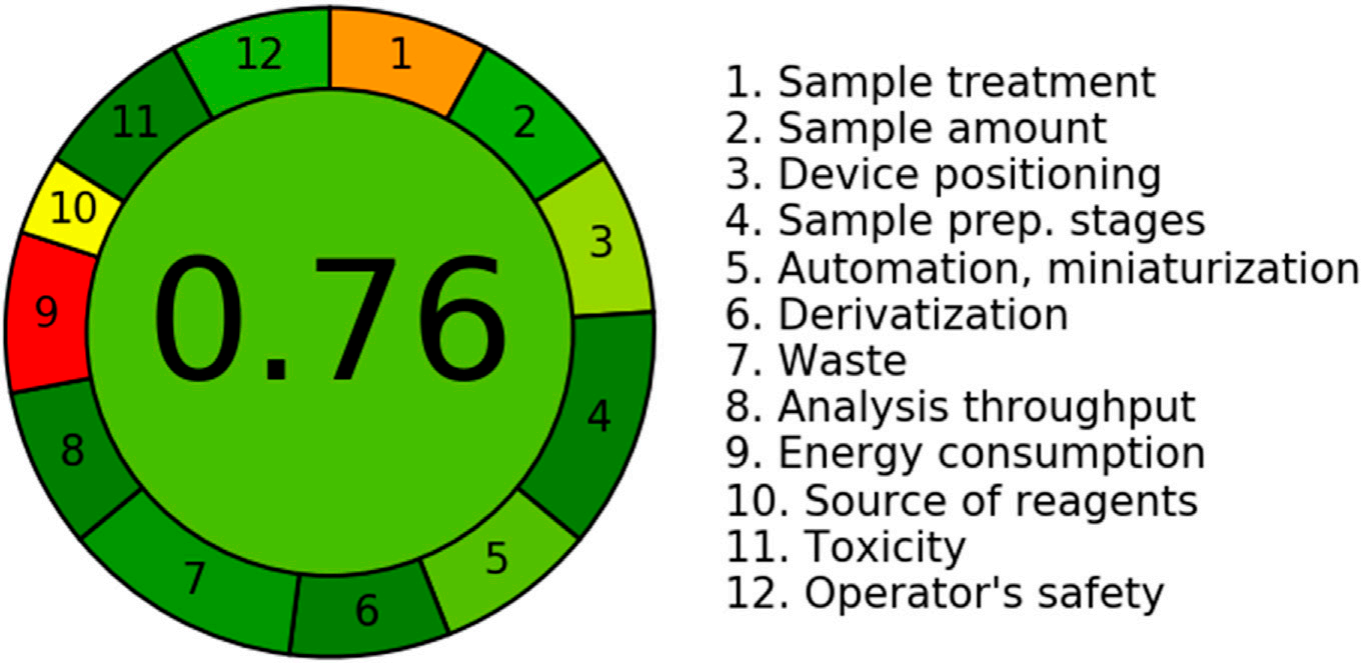
The application of AGREE program facilitated the presentation of the environmentally sustainable characteristics of the LC-MS/MS method. The results are visually shown by a circular diagram, which displays a diverse range of colors ranging from red (representing the lack of greenness approach) to dark green (showing the maximum degree of greenness). The aforementioned colors are representative of twelve unique characteristics, as depicted in the accompanying visual representation.

The assessment of the sustainability of the suggested LC-MS/MS technique was carried out utilizing the computer software AGREE, which encompasses all twelve aspects of Green Analytical Chemistry (GAC) (Pena-Pereira et al., 2020). The software allocates weights within the range of 0.0–1.0 to the various variables of the GAC system. The results are graphically depicted in a circular pattern that includes a wide range of colors, spanning from red to dark green, signifying twelve separate traits. Figure 5 illustrates the scaling curve of the LC-MS/MS technology in relation to its ecological sustainability. The scores corresponding to each of the twelve attributes are displayed in Table 5. The score of 0.76 was derived by evaluating numerous features of the prevailing approach, demonstrating the degree of sustainability that is related to the LC-MS/MS analytical procedure. A score nearing 1.0 indicates a methodology that exhibits a higher degree of ecological friendliness. The LC-MS/MS technique demonstrates a notable level of environmental sustainability when the eco-scale values fall within the range of 0.75–1.00.

**TABLE 5:** A report sheet has been developed for the UPLC-MS/MS approach in order to evaluate the environmental sustainability of the current methodology.

Measures	Score	Weight
1. It is advisable to employ direct methods of analysis so as to minimize the need for sample manipulation	0.3	2
2. The objectives of this research are to lower the amount of samples and decrease the total number of samples needed	0.82	2
3. If possible, it is recommended to conduct assessments in their original context, if feasible	0.66	
4. The amalgamation of analytical methodologies and operational processes possesses the capacity to effectively preserve energy resources and reduce the use of chemical substances	1.0	2
5. It is advisable to go for mechanized and reduced processes	0.75	3
6. It is advisable to refrain from employing the derivatization techniques	1.0	2
7. The prioritization of reducing a substantial quantity of analytical waste and implementing successful control measures for its disposal is of utmost importance	0.91	3
8. There exists a predominant tendency to the utilization of multi-analyte or multi-parameter methodologies as opposed to single-analyte techniques	1.0	2
9. Efforts must be made to reduce the consumption of energy	0.0	2
10. It is advisable to prioritize the consumption of reagents derived from sustainable sources	0.5	2
11. The prioritization of eliminating or substituting dangerous compounds is of paramount significance	1.0	3
12. There is a compelling need to enhance the safety standards for workers	0.8	2

This assessment is conducted by assigning individual scores based on the Green Analytical Chemistry (GAC) principles.

### 3.4 *In silico* assessment of CEP-37440 metabolic lability

**FIGURE 6:**
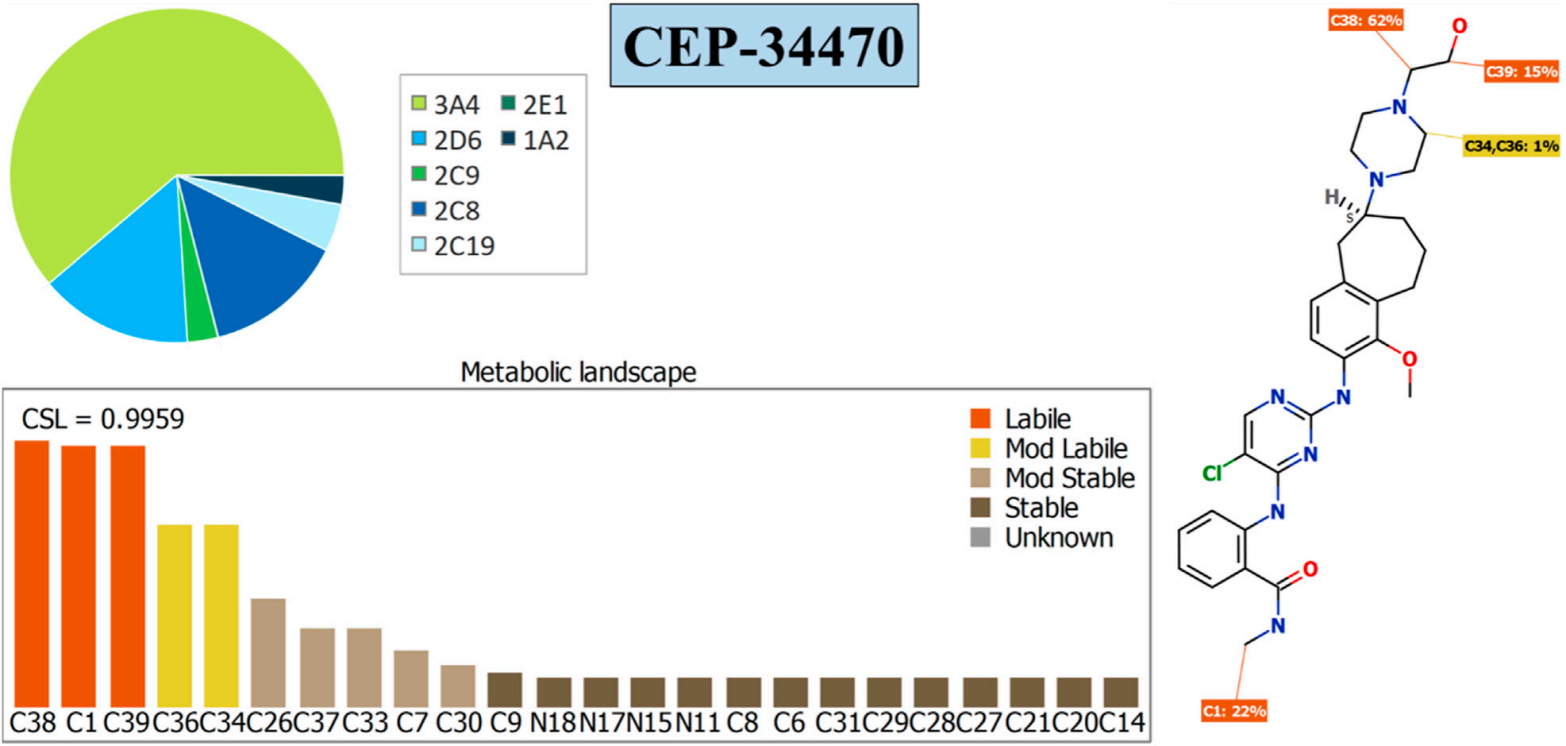
CSL value (0.9980) indicating the high lability of CEP-37440 to metabolism. The outcomes were evaluated utilizing the P450 module of the StarDrop’s software package.

The CSL value in the CEP-37440 metabolic landscape is used as an indication for the metabolic lability of the active sites inside the chemical structure of CEP-37440 relative to CYP3A4 enzymes. This information is visually represented in the pie chart. The assessment of CEP-37440s metabolic stability involved the utilization of the CSL value of 0.9959, as illustrated in Figure 6. To evaluate the metabolic stability of CEP-37440 during incubation with the active HLMs matrix, an LC-MS/MS approach was utilized. The metabolic lability of CEP-37440 is primarily influenced by the C38 (62%) and C39 (15%) positions in the ethanolamine group, as well as the C1 (22%) position in the N-methyl group. Additionally, a modest degree of metabolic instability (1%) was observed at the piperazine ring, specifically at carbon atoms C34 and C36. This assertion is substantiated by the robust connection observed between the metabolic lability and the CSL value of 0.9959, as illustrated in Figure 6. The aforementioned conclusion aligns with the outcomes obtained from the *in vitro* incubation experiments, that will be expounded upon in later parts.

### 3.5 *In silico* toxicity prediction of CEP-37440 using DEREK software

**FIGURE 7:**
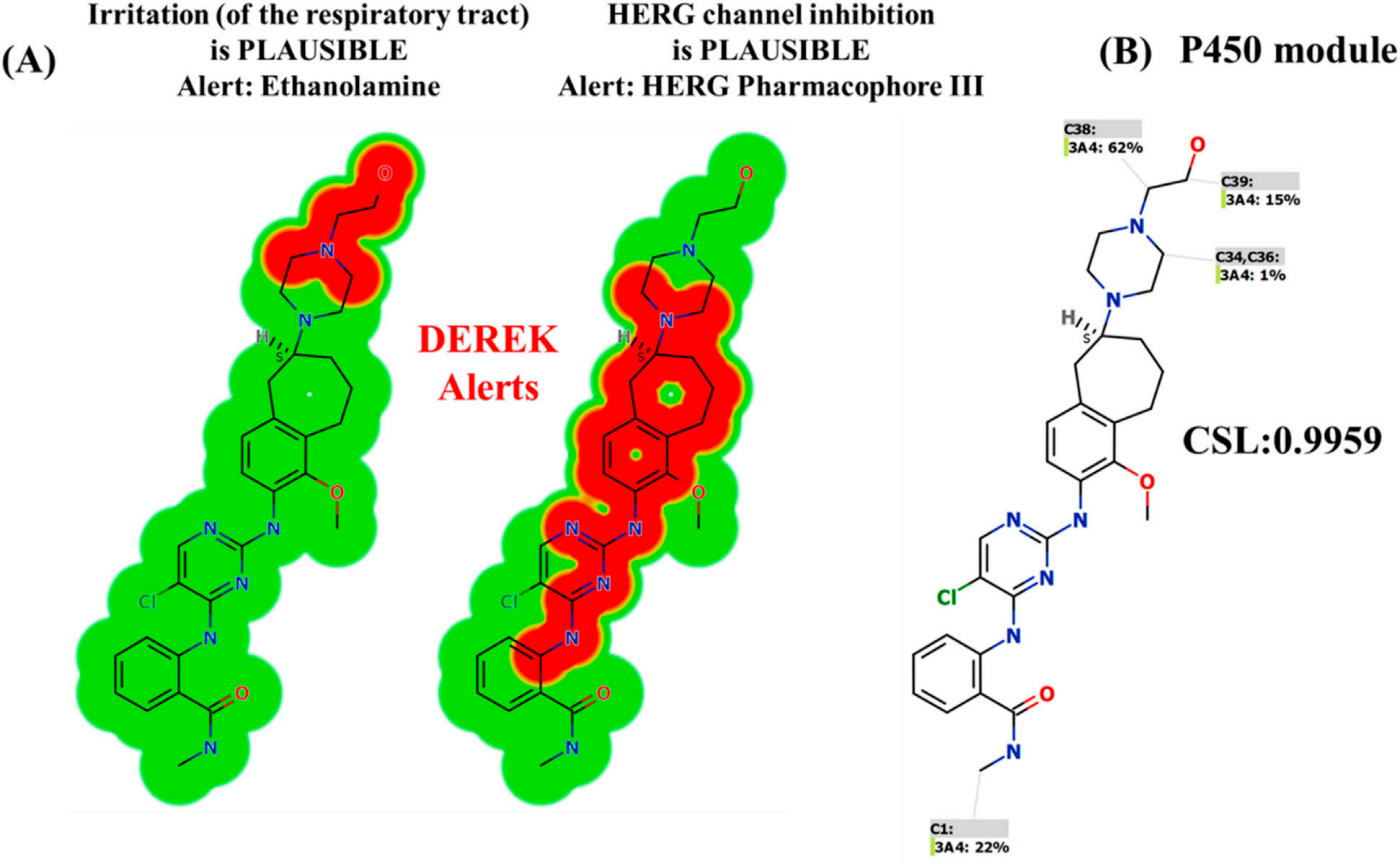
Structural alerts of CEP-37440 using DEREK software highlighted in red colour **(A)**. P450 metabolic stability of CEP-37440 indicating CSL at 0.9959 **(B)**.

Depending on the computational predictions and the information derived from scholarly sources, a compilation of plausible metabolites can be generated. The toxicity assessment of CEP-37440 was conducted using the DEREK software *in silico* (Figure 7A). The compound CEP-37440 exhibits structural warnings, as illustrated in Figure 3. These alerts are responsible for the hypothesized effects, namely, irritation of the respiratory tract and inhibition of the human ether-a-go-go-related gene (HERG) channel. The irritation of the respiratory tract is attributed to the presence of ethanol amine, while the inhibition of the HERG channel is associated with the presence of HERG pharmacophore III. The outcomes of this study reveal that the observed hazardous side effect may be attributed to the significant metabolic instability observed at the ethanol amine group. Minor structural alterations to the ethanolamine moiety or substitution of the group in drug design have the potential to enhance the metabolic stability and safety profile of novel derivatives in comparison to CEP-37440 (Figure 7B).

### 3.6 *In vitro* metabolic incubations of CEP-37440 with HLMs matrix

**FIGURE 8:**
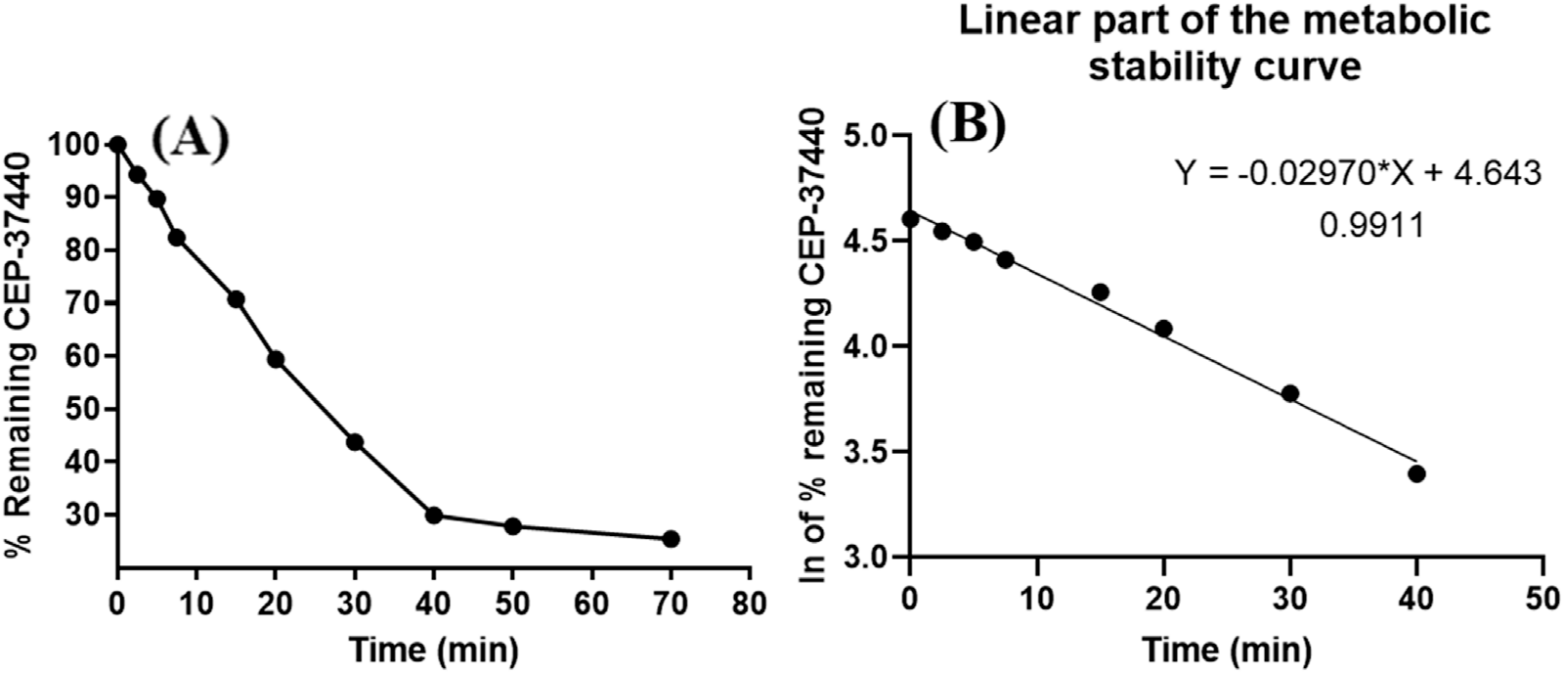
**(A)** CEP-37440 metabolic stability curve in HLMs matrix; **(B)** the logarithm (ln) curve viewing the regression equation of the linear part of the CEP-37440 metabolic stability curve.

The negative control sample exhibited no statistically significant reduction in the concentration of CEP-37440. To assess the metabolic stability of CEP-37440 *in vitro*, the compound CEP-37440 was employed at a concentration of 1 μmol/mL in the *in vitro* experiments utilizing HLMs. The selection of this particular concentration was made in order to maintain a value lower than the Michaelis-Menten constant (Darwish et al., 2018). The choice was made with the intention of maintaining a consistent linear relationship among the time frame of the *in vitro* incubation period and the rate at which CEP-37440 is metabolized. To mitigate non-specific protein binding, a concentration of 1 mg/mL of HLMs protein was utilized. The initial metabolic stability curve of CEP-37440 is depicted in Figure 8A. The *x*-axis shows specific intervals of time for the quenching process spanning 0–70 min, while the *y*-axis reflects the associated remaining ratio of CEP-37440. The linear section in the previous curve was chosen from the time interval of 0–40 min. The aim of this experiment was to generate a curve that depicts the relationship between the time points of metabolic incubation, spanning from 0 to 40 min, and the natural logarithm of the residual ratio of CEP-37440. The manifestation of this phenomenon is readily illustrated in Figure 8B. According to the research findings, it was observed that the metabolic rate of CEP-37440 had a slope of −0.0297, as evidenced by the linear equation y = −0.0297x + 4.643. The *r* ^2^ value of 0.9911 was observed for this equation, as indicated in Table 6. The calculation of the *in vitro* t_1/2_ involves dividing the natural logarithm of 2 by the slope. Therefore, the *in vitro* t_1/2_ was found to be 23.24 min. The clearance of CEP-37440 Cl_int_ was determined to be 34.74 mL/min/kg through calculation. According to the scoring approach devised by McNaney et al. (2008), it has been demonstrated that CEP-37440 has a modest degree of metabolic clearance. Several computational software applications, including Cloe PK and simulation tools, can be employed to predict the *in vivo* pharmacokinetics of CEP-37440 (Leahy, 2006).

**TABLE 6:** Metabolic stability of CEP-37440.

Time points (Min.)	Mean^a^ (ng/mL)	X^b^	LN X	Linearity parameters
0.00	466.85	100.00	4.61	Regression line equation: y = −0.0297x + 4.643
2.50	425.67	91.18	4.51	
5.00	400.46	85.78	4.45	*R* ^2^ = 0.9911
7.50	366.10	78.42	4.36	
15.00	305.41	65.42	4.18	Slope: −0.0297
20.00	248.27	53.18	3.97	
30.00	174.84	37.45	3.62	t_1/2_: 23.24 min and
40.00	117.41	25.15	3.22	Cl_int_: 34.74 mL/min/kg
50.00	101.54	21.75	3.08	
70.00	100.05	21.43	3.06	

^a^ Average of three readings.

^b^ X: Average of the % residual concentration of CEP-37440 in three readings.

## 4 Conclusion

**FIGURE 9:**
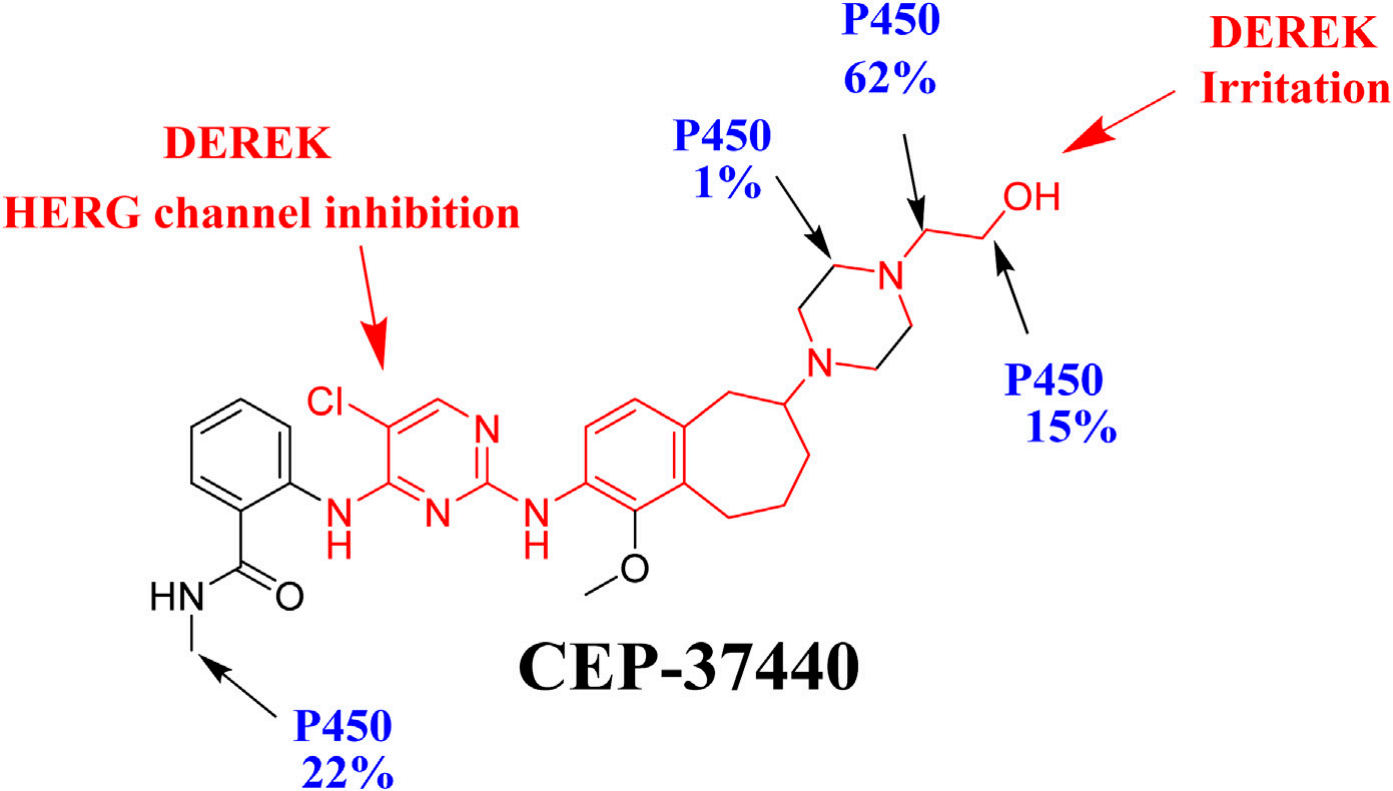
CEP-37440 metabolic lability curve (blue color) using P450 software and CEP-37440 DEREK toxicity predictions (red color) indicating that ethanol amine moiety is accountable for the metabolic instability of CEP-37440.

The present research reports the development and validation of a liquid chromatography-tandem mass spectrometry (LC-MS/MS) method for estimating the concentration of CEP-37440 in the matrix of human liver microsomes (HLMs). The technique outlined above was subsequently utilized to determine the metabolic stability of CEP-37440. The application of LC-MS/MS technology displayed several beneficial attributes, including enhanced sensitivity, specificity, environmental sustainability, and efficient recovery of CEP-37440 and IS from the HLMs matrix. The extraction process of choice was the protein precipitation protocol. The implementation of a reduced flow rate of 0.4 mL/min, accompanied by a shorter elution time of 2 min, has resulted in the current LC-MS/MS approach being environmentally sustainable. Through the usage of the AGREE program, an evaluation of the environmental impact has been conducted, revealing that the LC-MS/MS approach possesses environmentally friendly attributes and can be considered appropriate for the routine analysis of CEP-37440 without any detrimental consequences on the environment. The results derived from the metabolic P450 software were verified by comparing them to the results obtained from *in vitro* HLMs incubation assays. The findings from the investigation on metabolic stability demonstrate that CEP-37440 possesses a half-life (t_1/2_) of 23.24 min and a moderate clearance (Cl_int_) of 34.74 mL/min/kg. These results suggest that CEP-37440 can be categorized as a pharmaceutical agent with a moderate extraction ratio. Consequently, it is postulated that the administration of CEP-37440 to patients may not lead to the accrual of dosages within the human organs. According to *in silico* P450 metabolic and DEREK software, minor structural alterations to the ethanolamine moiety or substitution of the group in drug design have the potential to enhance the metabolic stability and safety profile of novel derivatives in comparison to CEP-37440 (Figure 9). Potential future research could be conducted using the current methodology, which involves the utilization of *in vitro* metabolic incubations and *in silico* software tools. The utilization of these methodologies is crucial for the progression of innovative pharmaceutical development, specifically with regard to augmenting metabolic stability. The usefulness of utilizing various *in silico* software strategies in conserving resources and minimizing effort was proved by the findings acquired from *in vitro* incubation studies and *in silico* software of CEP-37440.

## Data Availability

The original contributions presented in the study are included in the article/supplementary material, further inquiries can be directed to the corresponding author.
